# Novel Food Safety Evaluation: Potentially Toxic Elements in *Acheta domesticus* (House Cricket) Reared on Seaweed-Enriched Diets

**DOI:** 10.3390/molecules30193958

**Published:** 2025-10-02

**Authors:** Behixhe Ajdini, Irene Biancarosa, Silvia Illuminati, Anna Annibaldi, Federico Girolametti, Matteo Fanelli, Lorenzo Massi, Cristina Truzzi

**Affiliations:** 1 Department of Life and Environmental Sciences, Università Politecnica delle Marche, Via Brecce Bianche, 60131 Ancona, Italy; b.ajdini@staff.univpm.it (B.A.); s.illuminati@staff.univpm.it (S.I.); a.annibaldi@staff.univpm.it (A.A.); f.girolametti@staff.univpm.it (F.G.); 956820@stud.unive.it (L.M.); 2 Department of Marine Biotechnology, Stazione Zoologica “Anton Dohrn”, Fano Marine Center, Viale Adriatico 1-N, 61032 Fano, Italy; 3 Institute for Marine Biological Resources and Biotechnology (IRBIM), National Research Council (CNR), Largo Fiera della Pesca, 60125 Ancona, Italy; matteo.fanelli@irbim.cnr.it; 4 Department of Environmental Sciences, Informatics and Statistics, Università Ca’ Foscari Venezia, Via Torino 155, 30172 Mestre, Italy

**Keywords:** house cricket, novel food, seaweed, potentially toxic elements, risk assessment

## Abstract

In recent years, insects have emerged as a nutritious and eco-sustainable alternative food source, with the house cricket (*Acheta domesticus*, AD) recently authorized by the European Commission as a novel food. However, the presence of harmful substances in insects poses potential health risks. This study investigated the content of potentially toxic elements (PTEs) such as cadmium (Cd), arsenic (As), lead (Pb), mercury (Hg), nickel (Ni), chromium (Cr), and aluminium (Al) in *Acheta domesticus* fed diets enriched with graded levels of the red seaweed *Palmaria palmata* or the brown seaweed *Ascophyllum nodosum* in two feeding trials. Chemical analyses were carried out by graphite furnace atomic absorption spectrophotometry for all elements except Hg, which was analyzed by thermal decomposition amalgamation atomic absorption spectrometry. The results showed that PTE content in the diets was below the legal limits for feed. The PTEs in AD ranged (mg kg^−1^ dry matter) as follows: Cd (0.069 ± 0.005–0.127 ± 0.002), As (0.08 ± 0.01–0.36 ± 0.03), Pb (0.05 ± 0.01–0.12 ± 0.01), Hg (0.0065 ± 0.0002–0.0141 ± 0.0010), Ni (0.64 ± 0.06–1.20 ± 0.10), Cr (0.16 ± 0.02–0.58 ± 0.01), and Al (17 ± 2–61 ± 1). AD bioaccumulated As and Hg; however, the PTE levels remained below European Union food safety limits. The absence of non-carcinogenic risk for consumers suggests that AD fed seaweed-enriched diets are a safe, healthy, and low-chemical risk food for humans.

## 1. Introduction

Due to the increase in the world population, there has been a growing interest in new sources of protein in the past years [1]. Traditional animal farming is raising concerns regarding its impact on the environment and also on human health [2,3]. Therefore, given the large ecological footprint of traditional food production chains, it has become important to explore alternative food resources that could provide enough food for the growing population with only a minor impact on the environment [4,5]. Edible insects are considered promising novel food resources, thanks to their numerous advantages in terms of nutritional profile and sustainability [6]. They are rich in protein, essential vitamins, fat, micro and macro-minerals [7]. Moreover, insect production emits minimal amounts of greenhouse gases, provides a high percentage of edible mass, has a high feed-conversion efficiency, and requires little space, feed, and water [8].

When insects are intended to be used as a novel food, safety evaluations are important to ensure a safe product for consumers. Insects can accumulate both essential and non-essential elements from their diet. Essential elements, such as iron (Fe), zinc (Zn), copper (Cu), are required for normal physiological functions, whereas non-essential elements, including potentially toxic elements (PTEs) such as cadmium (Cd), arsenic (As), lead (Pb), mercury (Hg), nickel (Ni), chromium (Cr), and aluminum (Al) pose potential health risks, even at low concentrations [9]. Therefore, monitoring the presence of PTEs in edible insects is critical to ensure the safety of these products for humans. Pb, Cd, Hg, and As are regulated in both feed and food under European Regulations No. 1869/2019 [10] and No. 1987/2024 [11] regarding maximum levels of Ni and the amending regulation 915/2023 [12], respectively, whereas Cr and Al have not been fully assessed [13].

According to the European Food Safety Authority, the levels of contaminants in insects are significantly influenced by several factors, including the composition of the rearing substrate, the methods used for insect production and transformation, insect species, and the life-stage at which they are harvested [6]. Studies on PTEs contamination in insects (and derived products) are still poor [14,15,16,17,18,19]. Various studies have demonstrated that metals can occur naturally in insects but are often transferred from substrates on which they are reared [15,16,18,19,20].

The house cricket (*Acheta domesticus*, AD) has recently been approved by the European Commission as novel food (EU 2022/188) [21]. This species is characterized by a high protein content (55–70% on dry matter, DM), with a good index of essential amino acids [22,23,24,25].

In the current study, AD were fed diets enriched with graded levels of the red seaweed *Palmaria palmata* [24] and the brown seaweed *Ascophyllum nodosum* [25] in two separate feeding trials. These species were selected based on their nutritional composition and potential as eco-sustainable feed ingredients, aiming to improve the nutritional composition of AD. They are naturally known to be rich in minerals, vitamins, micro–macro nutrients, and omega-3 fatty acids like eicosapentaenoic acid [26]. Their inclusion in insects’ rearing substrate can improve the insects’ nutritional profile for feed and food purposes [16,24,25,27,28,29,30,31]. Seaweed biomass was incorporated into the diet, rather than provided directly to the insects, to ensure a homogeneous diet with the standard substrate across different levels of inclusion. Previous studies have shown that PP supplementation slightly affects the nutritional composition of AD, increasing the unsaturated fatty acids and leading to the introduction of a health-promoting fatty acid, eicosapentaenoic acid (EPA) [24], while AN supplementation was shown to enrich AD with monounsaturated fatty acids and omega-3 long chain polyunsaturated fatty acids (n-3 LC-PUFA), including EPA and docosahexaenoic acid, even though the overall n3 LC-PUFAs levels remained relatively low [25]. Despite its nutrients, seaweed also naturally contains PTEs, with concentrations varying between species as well as seasonal and geographical origin [32,33,34]. PTEs can therefore enter the food chain when seaweed biomass is used in insect rearing substrate, leading to potential risks to human health [16,19,28,31,35].

Despite the growing interest in edible insects and the use of alternative feed ingredients, the transfer of PTE from seaweed-enriched substrates to house crickets has not yet been investigated. The present study aimed to evaluate the effects of using seaweed-enriched diets for AD-rearing on the potential transfer of PTEs (Cd, As, Pb, Hg, Ni, Cr, and Al) from the substrates to the insects. Moreover, a health risk assessment was conducted to evaluate the safety of AD for human consumption.

## 2. Results

### 2.1. Potentially Toxic Elements Content in Seaweed and Diets

PTE content in both seaweed and diets is reported in Table 1. Table S1 shows the acceptable limit of PTE content in diets with a moisture of 12%, as reported in the legal limits for complete feed in Commission Regulation No. 1869/2019 [10], shown in comparison with our results. The seaweed biomasses of *Palmaria palmata* (PP) and *Ascophyllum nodosum* (AN) showed a similar content of Cd (~0.3 mg kg^−1^ DM) and Cr (range: 1.53 (AN)–1.75 (PP) mg kg^−1^ DM), whereas PP had from a three to fivefold higher content of Pb, Ni, and Al, a fifty-time lower content of As and a fivefold lower content of Hg, compared to AN.

In the diets, the element with the highest content was Al, reaching up to ~400 mg kg^−1^ DM, while the element with the lowest content was Hg, in the order of µg kg^−1^ (range 2.4–28 µg kg^−1^). In general, the PTE content of the diets (Table 1) was influenced by adding seaweed biomass. In trial 1, with increasing PP inclusion in the diet, a statistically significant increase was evidenced for As (*p* = 0.0073), Pb (*p* = 0.0006), and Hg (*p* = 0.0004), while a statistically significant decrease occurred for Ni (*p* = 0.0451). In trial 2, when increasing the percentage of AN included in the diet, a statistically significant increase was evidenced for Cd (*p* = 0.0001), As (*p* = 0.0011), and Hg (*p* = 0.0001), while a significant decrease occurred for Al (*p* = 0.0004). Moreover, a statistically significant decrease in Pb (*p* = 0.0103), Ni (*p* = 0.0303), and Cr (*p* = 0.0045) was evidenced in AN40 diets with respect to the ctrl diet (Table 1).

### 2.2. Potentially Toxic Element Content in Acheta domesticus

**Figure 1:**
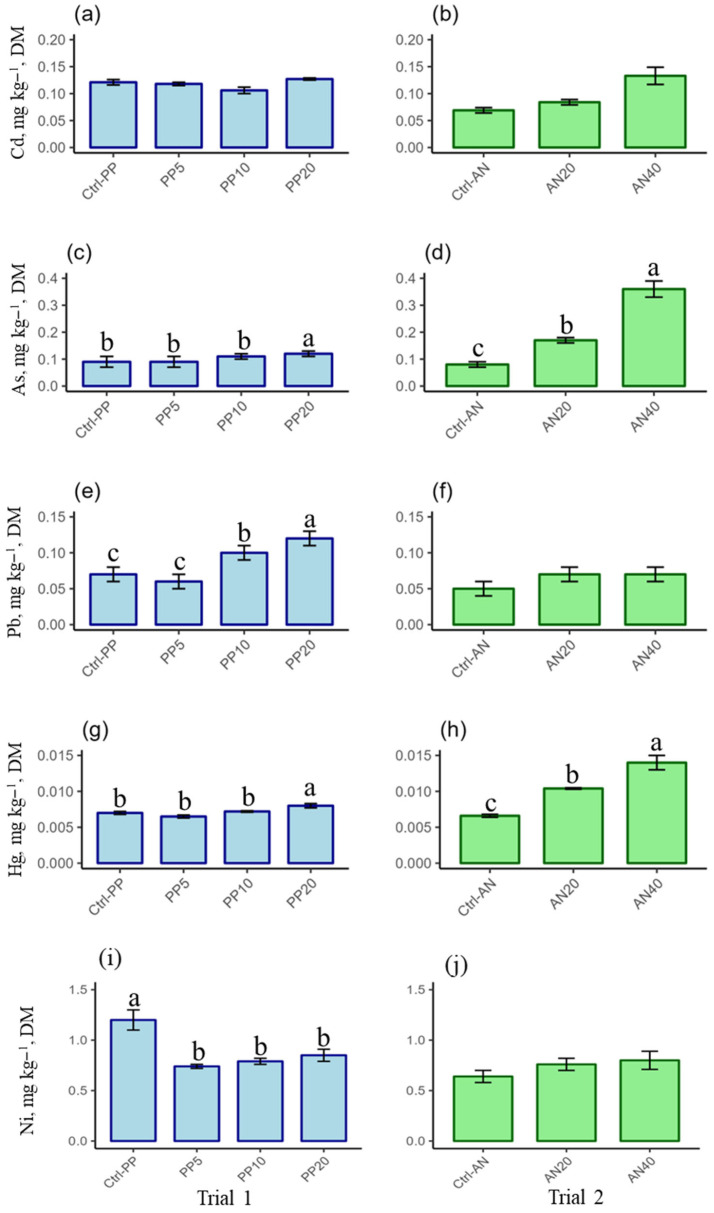
Cd, As, Pb, Hg, and Ni content in *Acheta domesticus* fed *Palmaria palmata*-enriched diets (trial 1) and *Ascophyllum nodosum*-enriched diets (trial 2). (**a**) Cd content in AD fed *Palmaria palmata*-enriched diets (trial 1); (**b**) Cd content in AD fed *Ascophyllum nodosum*-enriched diets (trial 2); (**c**) As content in AD fed *Palmaria palmata*-enriched diets (trial 1); (**d**) As content in AD fed *Ascophyllum nodosum*-enriched diets (trial 2); (**e**) Pb content in AD fed *Palmaria palmata*-enriched diets (trial 1); (**f**) Pb content in AD fed *Ascophyllum nodosum*-enriched diets (trial 2); (**g**) Hg content in AD fed *Palmaria palmata*-enriched diets (trial 1); (**h**) Hg content in AD fed *Ascophyllum nodosum*-enriched diets (trial 2); (**i**) Ni content in AD fed *Palmaria palmata*-enriched diets (trial 1); (**j**) Ni content in AD fed *Ascophyllum nodosum*-enriched diets (trial 2). AD fed a control diet with the inclusion of 0% of *Palmaria palmata* (Ctrl-PP) and 0% of *Ascophyllum nodosum* (Ctrl-AN); AD fed diets enriched with 5% (PP5), 10% (PP10), and 20% (PP20) of *P. palmata*; AD fed diets enriched with 20% (AN20) and 40% of *A. nodosum* (AN40). Different letters indicate statistically significant differences between groups of the same trial (*p* < 0.05). Values are presented as mean ± SD (*n* = 9).

**Figure 2:**
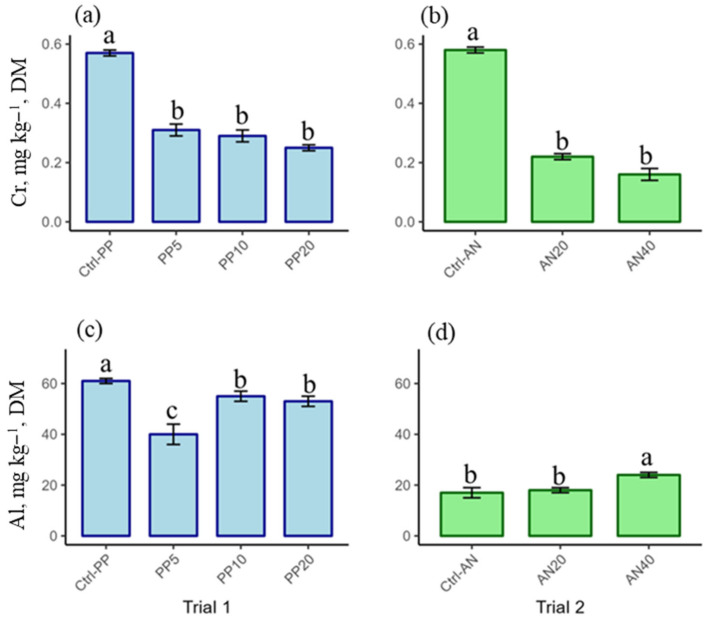
Cr and Al content in *Acheta domesticus* fed *Palmaria palmata*-enriched diets (trial 1) and *Ascophyllum nodosum*-enriched diets (trial 2). (**a**) Cr content in AD fed *Palmaria palmata*-enriched diets (Trial 1); (**b**) Cr content in AD fed *Ascophyllum nodosum*-enriched diets (Trial 2); (**c**) Al content in AD fed *Palmaria palmata*-enriched diets (Trial 1); (**d**) Al content in AD fed *Ascophyllum nodosum*-enriched diets (Trial 2). AD fed a control diet with the inclusion of 0% of *Palmaria palmata* (Ctrl-PP) and 0% of *Ascophyllum nodosum* (Ctrl-AN); AD fed diets enriched with 5% (PP5), 10% (PP10), and 20% (PP20) of *P. palmata*; AD fed diets enriched with 20% (AN20) and 40% of *A. nodosum* (AN40). Different letters indicate statistically significant differences between groups of the same trial (*p* < 0.05). Values are presented as mean ± SD (*n* = 9).

PTE content in AD is shown in Figure 1 for Cd, As, Pb, Hg, and Ni (regulated PTEs) in Figure 2 for Cr and Al (non-regulated PTEs). Table 2 shows the bioaccumulation factor of PTEs in AD, calculated on a DM basis.

Cadmium. In trial 1 (Figure 1a), Cd content showed a mean value of 0.12 ± 0.01 mg kg^−1^ DM (min–max, 0.106 ± 0.006–0.127 ± 0.002 mg kg^−1^ DM); no statistically significant differences were observed between experimental groups. In trial 2 (Figure 1b), on the contrary, Cd content increased significantly (*p* = 0.0001) with increasing inclusion of AN in the diet, passing from 0.069 ± 0.005 mg kg^−1^ DM in AD fed the ctrl diet to 0.13 ± 0.02 mg kg^−1^ DM in AD fed AN40. A statistically significant positive linear correlation (r = 0.9204, *p* = 0.0033) was verified between Cd content in AD and in the diets. The BAF did not show statistically significant differences between AD groups and trials, with an average value of 1.00 ± 0.09 (min–max, 0.86 ± 0.05–1.1 ± 0.2) (Table 2).

Arsenic. In trial 1, the inclusion of PP in the diets led to a statistically significant increase (*p* = 0.0059) of As in AD fed the highest seaweed inclusion (PP20) (0.12 ± 0.01 mg kg^−1^ DM) (Figure 1c) compared to AD fed the ctrl diet (0.09 ± 0.02 mg kg^−1^ DM). In trial 2, the increasing inclusion of AN in the diets led to a statistically significant increase in As in AD (*p* = 0.0073) which ranged from 0.08 ± 0.01 mg kg^−1^ DM in AD fed the ctrl diet to 0.36 ± 0.03 mg kg^−1^ DM in AD fed the AN40 diet (Figure 1d). A statistically significant positive linear correlation (r = 0.961, *p* = 0.0005) between As content in AD and in the diets was found. In trial 1, the BAF (mean value 1.5 ± 0.7), decreased significantly (*p* = 0.0020) in AD fed the PP20 diet compared to the other groups (Table 2). In trial 2, the BAF decreased significantly (*p* = 0.0402) in AD fed AN20 and AN40 (0.32 ± 0.03 and 0.40 ± 0.04, respectively) compared to AD fed the ctrl diet (1.5 ± 0.2).

Lead. In trial 1, Pb content increased significantly (*p* = 0.0356) when increasing the inclusion of PP in the diet (0.12 ± 0.01 mg kg^−1^ DM in AD fed PP20 diet, and 0.10 ± 0.01 mg kg^−1^ DM in AD fed PP10 diet) compared to AD fed the ctrl diet (0.07 ± 0.01 mg kg^−1^ DM) (Figure 1e). In trial 2, Pb content slightly increased from 0.05 ± 0.01 mg kg^−1^ DM in AD fed the ctrl diet to 0.07 ± 0.01 mg kg^−1^ DM in AD fed AN20 and AN40 diets, but no statistically significant differences were observed between groups (Figure 1f). A statistically significant positive linear correlation (r = 0.8540, *p* = 0.014) between Pb content in AD and in the diets was evidenced. The BAF value for Pb was lower than 1 for all AD groups, with a mean value of 0.17 ± 0.06 and of 0.23 ± 0.07 in trials 1 and 2, respectively (Table 2). In trial 1, BAF significantly decreased in AD fed PP-enriched diets compared to AD fed the ctrl diet (*p* = 0.0134), while in trial 2, the BAF significantly increased in AD fed AN40 diets compared to the other groups (*p* = 0.0347).

Mercury. Hg levels in trial 1 significantly increased from 0.007 ± 0.0002 mg kg^−1^ DM in ctrl AD to 0.0080 ± 0.0003 mg kg^−1^ DM in AD fed PP20 (Figure 1g) (*p* = 0.0075). In trial 2, Hg content significantly increased (*p* = 0.0001) in AD when increasing the inclusion of AN in the diets (0.0104 ± 0.006 mg kg^−1^ DM and 0.0141 ± 0.0010 mg kg^−1^ DM in AD fed AN20 and AN40 diets, respectively) with respect to the ctrl AN (0.0066 ± 0.0002 mg kg^−1^ DM) (Figure 1h). A statistically significant positive linear correlation (r = 0.9944, *p* ≤ 0.0001) between the Hg content in AD and in the diets was found. BAF for Hg was higher than 1 for all the experimental groups (Table 2). In particular, it showed the highest value in AD fed the ctrl diets (2.88 ± 0.07 and 2.60 ± 0.11 in trials 1 and 2, respectively), then it significantly decreased both in AD fed PP-enriched diets (*p* = 0.0003) and in AD fed AN-enriched diets (*p* = 0.0007).

Nickel. In trial 1, AD fed PP-enriched diets showed a significantly lower Ni content (*p* = 0.0001) (range 0.74–0.85 mg kg^−1^ DM), compared to AD fed the ctrl diet (1.20 ± 0.10 mg kg^−1^ DM) (Figure 1i). In trial 2, no statistically significant differences were observed between experimental groups (mean value, 0.73 ± 0.08 mg kg^−1^ DM, min–max, 0.64 ± 0.06–0.80 ± 0.09 mg kg^−1^ DM); (Figure 1j). No statistically significant correlation was found between Ni content in AD and their diets. BAF was <1 in both trials, with an overall average value of 0.15 ± 0.04. In trial 1, the BAF of AD fed the PP5 and PP10 diets was significantly lower with respect to AD fed the ctrl diet (*p* = 0.0092), while in trial 2, the BAF of AD fed AN40 diets was significantly higher compared to AD fed ctrl diets (*p* = 0.0275) (Table 2).

Chromium. In trial 1, AD fed PP-enriched diets showed a significantly lower Cr content (range 0.25–0.31 mg kg^−1^ DM) (*p* = 0001) compared to AD fed the ctrl diet (0.57 ± 0.01 mg kg^−1^ DM) (Figure 2a). In trial 2, Cr showed a significantly (*p* = 0.0001) lower content in AD fed AN-enriched diets (0.16 ± 0.02 and 0.22 ± 0.01 mg kg^−1^ DM in AD fed AN40 and AN20 diets, respectively) compared to AD fed the ctrl diet (0.58 ± 0.01 mg kg^−1^ DM) (Figure 2b). No statistically significant correlation (r = 0.1966, *p* = 0.6727) between Cr content in AD and in their diets was found. BAF value for Cr was far below 1, with an average value of 0.15 ± 0.01 for the Ctrl groups, and a mean value of 0.06 ± 0.01 for AD fed seaweed-enriched diets, which showed significantly lower values compared to their respective control groups (*p* < 0.0001 in trial 1, *p* = 0.0001 in trial 2) (Table 2).

Aluminum. This element showed the highest content with respect to the other considered PTEs. Compared to AD fed the ctrl diet in trial 1 (61 ± 1 mg kg^−1^ DM), AD fed PP-enriched diets showed a significantly lower Al content (*p* = 0.0141) (from 40 to 55 mg kg^−1^ DM) (Figure 2c). In trial 2, Al content in AD fed AN40 (24 ± 1 mg kg^−1^ DM) showed a statistically significant increase (*p* < 0.0001) compared to the other two groups (17 ± 2 and 18 ± 1 mg kg^−1^ DM for AD fed the ctrl and AN20 diets, respectively). A statistically significant correlation (r = 0.9094, *p* = 0.0045) between Al content in AD and in the diets was demonstrated. BAF for Al was far below 1, with an average value of 0.13 ± 0.02 for trial 1, and a mean value of 0.06 ± 0.02 for trial 2 (Table 2).

### 2.3. Health Risk Assessment

Table 3 shows the legal limit of PTEs in mg kg^−1^ WW [11,12,21] and their content in ADs from trials 1 and 2. As, Pb, and Hg content was far below their respective legal limits, with Hg two orders of magnitude lower, As about ten times lower, Pb from three to tenfold lower, and Ni about a hundred times lower. Cd content, while below the legal limit of 0.05 mg kg^−1^ WW for meat, was within the same order of magnitude. Cr and Al showed higher concentrations than the normed PTEs, but currently, no legal limits are established for them.

In this study, the risk assessment of PTE exposure for humans was carried out by calculating the values of the Hazard Quotient (HQ) (Table 4) and of the Hazard Index (HI) (Table 5) for each PTE. The HQ values were (mean ± SD): Cd, 0.008 ± 0.002; As, 0.005 ± 0.004; Pb, 0.0016 ± 0.0005; Hg, 0.0010 ± 0.0006; Ni, 0.0031 ± 0.0007; Cr, 0.008 ± 0.005; Al, 0.014 ± 0.007. The mean value of HI, obtained from the average of the HI values calculated for each experimental group, was 0.04 ± 0.01 (min–max, 0.026–0.056). Both the indices were well below 1. The contribution of the HQ for each element to the HI value was (in percentage): Al (33.6%), Cd (20.2%), Cr (19.7%), As (12.0%), Ni (7.8%), Pb (4.1%), Hg (2.6%).

## 3. Discussion

The consumption of insects requires a comprehensive assessment of the chemical risk profile associated with their use as food for humans. The levels of chemical elements in insects vary based on factors like insect type, growth stage, and rearing substrate [36,37,38]. Among these factors, the safety of the rearing substrate is particularly crucial when breeding insects. To the best of our knowledge, only Ajdini et al. [24,25] have conducted research on the use of seaweed as an ingredient for AD mass rearing, focusing on the performance parameters and nutritional aspects, while this study is the first to specifically assess the potential risk associated with the use of seaweed-enriched diets for AD.

### 3.1. Potentially Toxic Elements in Seaweed and Diets

The seaweed biomass and diets in this study showed a highly variable content of PTEs, ranging from a few μg kg^−1^ DM for Hg to hundreds of mg kg^−1^ DM for Al. Concerning PTE content in PP and AN were found in this study; this is consistent with the literature data on these species [39,40,41,42,43], but they highlight the substantial variability reported for seaweeds of the same species. This variability is likely related to the different contents of the studied elements that are present in the area where the seaweed is harvested, as well as the season, as noted in previous studies [42,44,45,46].

In our study, the PTE content in the two ctrl diets from trials 1 and 2 was generally similar, except with higher Cd levels in trial 1 with respect to trial 2. Since these diets consist primarily of plant-based ingredients, Cd content can be influenced by factors such as soil type, seasonal variations, and fertilizers or pesticides [42,44,45,46]. There are only a few studies in the literature reporting on PTE concentrations in the diets for farmed AD [47,48]. Pastell et al. [47] investigated three vegetable-based diets supplemented with vitamins for AD mass rearing and determined the concentrations of Cd, As, Pb, Ni, and Cr in those diets. Moreover, Jucker et al. [48] evaluated the use of maize distiller in hen diets, determining the contents of Pb, Cd, and As contained therein. Compared to the findings of Pastell et al. [47], the diets in these two trials showed comparable contents of Cd, As, and Pb and higher contents of Ni and Cr. Moreover, Pb content was consistent with that reported in the maize distiller diet by Jucker et al. [48], but higher with respect to the hen diet, whereas the concentrations of Cd, and As were higher than in the diet proposed by Jucker et al. [48].

According to the Regulation No. 1869/2019/EU [10], the contents of Cd, Pb, As, and Hg in the diets were well below the legal limits established for complete feed with 12% moisture, i.e., Cd, 0.5 mg kg^−1^ WW, Pb, 5 mg kg^−1^ WW, Hg, 0.1 mg kg^−1^ WW, and As, 2 mg kg^−1^ WW. Therefore, the seaweed-enriched diets are considered safe as regards the chemical risk and suitable for house cricket mass rearing. Currently, there are no legal limits for Cr, Ni, and Al, but given their toxicity and their presence in the diets from this study, as well as in previous studies [19], establishing limits is recommended for insect safety.

### 3.2. Potentially Toxic Elements in Acheta domesticus

A comparison of the results in this study with the content of the PTEs detected in AD fed different diets as reported in the literature is shown in Table 6.

Cadmium (Cd) is a carcinogenic element that affects multiple tissues [54] and can accumulate in the animal body for long periods before being fully eliminated [55]. The presence of this element is often reported in different insect species, such as black soldier fly larvae, grasshoppers, locusts, termites, and superworms [56]. In this study, Cd content in AD was up to one order of magnitude higher than the content reported in AD from the literature [47,49,50,51,52,53] (Table 6). In comparison to other insect species, AD contained less Cd than HI [16,19], and the edible grasshopper (*Oxya chinensis formosana*) [57], and more Cd than *T. molitor* [18]. Moreover, our data were in line with the results of the study by Elechi et al. [58] in HI grown on different diets.

Arsenic (As) is a mutagenic and carcinogenic element, considered one of the most prevalent causes of poisoning in animals [56]. As concentrations in AD were similar to those reported by Ververis et al. [52] but higher than the content found in the other studies reported in Table 6. Compared to other insect species, AD contained an As content similar to that found in HI [14,16,19], and in the *Oxya chinensis formosana* [57]. Conversely, other studies reported a lower As content in *T. molitor* larvae [18], in *Oxya chinensis formosana* [57], and in HI [17].

Lead (Pb) is a toxic element that has become a significant concern for the health of all organs in animals [59]. In this study, Pb content in AD was higher than that reported by Poma et al. [50] but comparable to the other studies reported in Table 6. Compared to other insect species, Pb content in AD fed seaweed-enriched diets was like that found in HI reared on algal substrates [16,19] but lower than *T. molitor* [18], and *Oxya chinensis formosana* [57].

Mercury (Hg) is considered a highly toxic element, particularly in its methylated form MeHg [59]. In the present study, Hg levels were lower than those found by Ververis et al. [52] and Kohler et al. [53] (Table 6). Compared to other insects, AD showed a lower Hg content than HI [19], *T. molitor*, *Oxya chinensis formosana* [57], *Jerusalem* and *Camel crickets* [60] and unspecified species [61].

Nickel (Ni) is a widely distributed element, being toxic in high concentrations [58]. Ni content in AD was higher than in the data obtained by Collavo et al. [49], Poma et al. [50], and Pastell et al. [47], while it was partially comparable to Kosečková et al. [51] and Ververis et al. [52] (Table 6). Compared to other insect species, Ni content in AD was partially comparable to the content found in HI [19] and in *T. molitor* [18].

Chromium (Cr) is considered toxic and can lead to allergenicity and carcinogenicity in both humans and animals. Cr content in AD was partially comparable to Poma et al. [50], Kosečková et al. [51], and Pastell et al. [47], but lower than in the data obtained by Collavo et al. [49] (Table 6).

Aluminum (Al) is associated with Alzheimer’s disease [62]. Only a few studies have reported on Al content in AD, of which Collavo et al. [49] found lower Al content than in this study (Table 6). Compared to other insect species, Al content in AD was consistent with the content found in HI reported by Bessa et al. [63].

In the current research, we found that the developmental stage of AD at which the feeding experiment started and the exposure time could have influenced the PTE content. In trial 1, the algae-based diets were administered to crickets for 7 days, starting from 20 days post-hatching, whereas in trial 2, the diets were provided to crickets for 14 days, starting from 14 days post-hatching. The extended feeding period in trial 2, initiated at an earlier development stage, led to a more marked increase in Cd, As, Hg, and Al compared to the contents observed in crickets from trial 1. These data align with literature studies, which found that the developmental stage of insects, as well as the exposure time, may affect the presence and the levels of PTEs in insects and their accumulation patterns [56,64,65]. Other studies demonstrated a different PTEs content in the different life stages of insects: Sun et al. [66], reported a lower concentration of Ni in the pupae and adults of *Spodoptera litura* compared to the larvae; Diener et al. [15] confirmed a decline in Pb, Cd, and Zn content in HI during metamorphosis. Similarly, it was reported that there was a decrease in Cr content from the larval stage to the adult stage [65].

This study demonstrated a statistically significant correlation between diet and Cd, As, Pb, Hg, and Al content in AD, but not for Ni and Cr. For most elements, these results are consistent with previous studies applied to different insect species, such as Cd and Pb for HI and *T. molitor* [15,20,67]. The higher content of some PTEs found in AD in this study compared to the literature would therefore be mainly due to their levels in the administered diets. Ni content in the AD was not correlated with the content in the diet, consistent with the findings by Truzzi et al. [18,19] on HI and *T. molitor*, indicating no influence from diets on the insect content.

A BAF value greater than 1 for Hg and As indicated that AD are capable of bioaccumulating these elements, as demonstrated for other insect species, such as *T. molitor* and HI [18,19], and for *Locusta migratoria malinensis* and *Achrida chinensis* [68].

Results about Hg bioaccumulation indicated lower values compared to the range found in HI and *T. molitor* [18,19] and higher compared to the value (1.13) in *Locusta migratoria malinensis* and *Achrida chinensis*, reported by Zhang et al. [68].

The results of As bioaccumulation align with Pastell et al. [47], who reported a BAF value of 0.04 in AD fed three different diets, and with Truzzi et al. [18], who reported a BAF below one in HI larvae and *T. molitor*. Conversely, Bessa et al. [63] reported a BAF > 1 in some HI experiments, indicating species-specific bioaccumulation abilities, as well as the influence of the diet. In this study, we found that increasing levels of seaweed in the diet, which significantly increased Hg and As content in the diet, led to a reduction in their respective BAF values in AD. For Hg, the BAF remained higher than one in all groups, whereas for As, it decreased below one in AD fed AN-enriched diets. These results suggested a reduced Hg and As bioavailability in seaweed, as already observed in the study by Truzzi et al. [19]. The low bioavailability of As in algae could be attributed to the high proportion of non-bioavailable organic arsenic species, such as arsenosugars, which account for approximately 90% of the total arsenic content [69]. Regarding Hg, seaweed predominantly accumulates mercury in its inorganic form, rather than as methylmercury, the more bioavailable and toxic form [70,71].

The elements Cd, Pb, Ni, Cr, and Al showed a BAF value of less than one in all AD treatment groups. In particular, the BAF nearest to one for Cd was consistent with the BAF of AD found by Pastell et al. [47] and of *T. molitor* [18,20]. Conversely, Truzzi et al. [19], Bessa et al. [63], and Elechi et al. [58] reported BAF values greater than one for HI larvae, highlighting a different ability to bioaccumulate Cd for different insect species. BAF values lower than one for Pb were consistent with Pastell et al. [47] and Van Der Fels-Klerx et al. [67] for HI larvae, and Zhang et al. [68] for *L. migratoria*. Pb bioaccumulation is species-dependent; in fact it has been demonstrated for HI [19,66], *T. molitor* [18], and grasshoppers [72], but not for locusts [68]. However, in the studies by Elechi et al. [58], Purschke et al. [17], and Tschirner and Simon [73], a BAF > 1 was recorded in half of the experimental groups of HI prepupae, suggesting that not only the species, but also the insect diet may influence the bioaccumulation of Pb. This can be explained by the fact that Pb content depends on its levels in the ingredients of the diet, but its bioavailability does not necessarily correlate with its content. A BAF lower than 1 for Ni confirmed that it was not bioaccumulated by AD, as found by Pastell et al. [47] (average BAF value 0.352) and Truzzi et al. [18,19] in HI and *T. molitor*. For Cr we found a BAF value below one, confirming no bioaccumulation. Pastell et al. [47] reported a BAF ranging from 0.37 to 1.6 and 3.96 for crickets fed three different diets, based on the bioavailability of this element in the ingredients of the standard diet used. Similar observations were reported by Bessa et al. [63] and Elechi et al. [58] in the prepupae of *Helicoverpa armigera* only in specific test groups fed with different types of diets. Finally, the BAF value for Al of less than one was consistent with Bessa et al. [63] for HI.

In terms of the regulatory compliance, the EU Regulation 1987/2024 of 30 July 2024 [11], as regards maximum levels of nickel in foodstuffs, and amending EU Regulation 915/2023 of 25 April 2023 [12], which establishes maximum levels for certain contaminants in foodstuffs, does not currently support maximum PTEs limits for the safe use of house crickets. However, the Commission Implementing Regulation (EU) 2022/188 of 10 February 2022 [21], which authorized the placing-on-the-market of AD, specified a content of Cd and Pb (≤0.05 and ≤0.06 mg kg^−1^, respectively) in frozen, dried and powdered AD. According to the allowable limits set out for products such as meat, rice, and fish and the levels proposed by the EU adopted on 10 February 2022, the legislated contaminants Cd, As, Pb, and Hg in AD fed seaweed-enriched diets were below the maximum limits and fall within the safe levels. Elements such as Cr and Al require particular attention, as they are not currently regulated for PTE content in either food or feed, and studies on their content in insects remain scarce. In contrast, Ni has established maximum levels in food, but no specific regulation exists for its presence in feed. Given the levels of these elements in AD, and their potential toxicity, further studies are necessary to support the establishment of specific limits for their presence in both food and feed. Regarding the potential exposure risks from the studied elements, Al emerged as the most significant, contributing 33.6% to the HQ in relation to the HI. Nevertheless, both HQ and HI values remained below one, indicating the absence of non-carcinogenic risk for consumers.

## 4. Materials and Methods

### 4.1. Diets

The study consisted of two separate feeding trials as described in detail elsewhere [24,25]. In trial 1, AD were fed four diets: (i) the control diet (Ctrl-PP) consisted of a standard substrate made of 100% plant-based ingredients, primarily cereals, such as wheat meal, corn, soy-bean meal, and beet, supplied by the insect farm Nutrinsect SRL (Montecassiano, Italy); (ii) three experimental diets in which the red seaweed PP was added to the standard substrate at three different percentages: 5% (PP5), 10% (PP10), and 20% (PP20). In trial 2, crickets were fed three diets: (i) the control diet, (ii) two experimental diets with the brown seaweed AN added at 20% (AN20) and 40% (AN40). The seaweed biomasses were purchased from Ocean Harvest (Castle Court, Ireland). Additional details on the diet preparation are available elsewhere [24,25]. Prior to the feeding trials, samples of the seaweed biomass and diets were collected and stored at −20 °C for PTE analysis.

### 4.2. House Cricket Rearing

Both feeding trials were conducted at Nutrinsect SRL using established *A. domesticus* colonies. Each trial included three replicates per group, and standardized rearing conditions were maintained throughout the trials, including ad libitum water access, a 12:12 light-dark cycle, and controlled temperature (30 ± 2 °C) and relative humidity (50 ± 2%). The feeding trials with seaweed-enriched diets started at 20 days post-hatching in trial 1, and at 14 days post-hatching in trial 2 to test the capacity for accumulation of PTE in AD at different developmental times. Detailed information on the experimental set up and rearing conditions is available in Ajdini et al. [24,25]. The feeding period lasted 7 days in Trial 1 (from day 20 post-hatching to day 27 post-hatching) and 15 days in Trial 2 (from day 14 post-hatching to day 29 post-hatching). At the end of each trial, crickets fasted for 24 h before being sacrificed in an industrial blast chiller at −40 °C for 30 min and subsequently stored at −20 °C until further analysis. All procedures complied with Italian legislation and institutional guidelines, which do not require specific authorization for research involving invertebrates such as insects.

### 4.3. Chemical Analysis

All laboratory procedures were carried out in a clean room laboratory classified as ISO 14644-1 Class 6, with ISO Class 5 under laminar flow, including decontamination protocols for trace metal determination [74]. Sample treatment was as in Ajdini et al. [24,25]. Analyses were performed on three aliquots per sample (~0.5 g each) using an analytical balance (AT261, Mettler Toledo, Greifensee, Switzerland).

For the determination of Cd, As, Pb, Ni, Cr, and Al, lyophilized samples (−20 °C in vacuum, 0.2–0.01 mBar) (BUCHI Lyovapor L-200, BUCHI s.r.l, Milan, Italy) were microwave-digested (MARS-5, 1500 W, CEM, Mathews, NC, USA) using a mixture of 3 mL of a high-quality (65% *v*/*v*) nitric acid HNO_3_ and 3 mL of 30% *v*/*v* H_2_O_2_ hydrogen peroxide (Merck, Darmstadt, Germany). Concentration in WW was calculated by referring to the weight of the fresh sample before the lyophilization procedure.

Operational parameters were set according to Girolametti et al. [75]. Quantification was performed using an Agilent 240 Z AAS Atomic Absorption Spectrometer equipped with a graphite furnace (GTA 120 Graphite Tube Atomizer) (Agilent Technologies, Santa Clara, CA, USA) and Zeeman-effect background correction. Calibration was carried out using standard solutions for each element from Titrisol solutions (Merck, Darmstadt, Germany), the analytical parameters refer to the linearity of the method, and the LOD and LOQ were also measured [76].

Hg content was determined using thermal decomposition amalgamation atomic absorption spectrometry with a Direct Mercury Analyzer (DMA-1, FKV, Milestone, Sorisole, BG, Italy). Homogenized samples were directly weighed into quartz containers, and the analysis conditions were optimized following Girolametti et al. [77]. Hg quantification was performed using the calibration curve method and a liquid mercury stock standard solution with a concentration of 1 g L^−1^ (Merck, Darmstadt, Germany), diluted to prepare the standard solutions.

All analyses were carried out in triplicate. Analytical quality control was ensured using the certified reference material DORM-2 (Dogfish Muscle Certified Reference Material for Trace Metals, National Research Council of Canada, Ottawa, ON, Canada) (Table 7). The results were consistent with the certified values, while the low standard deviations confirmed the accuracy and repeatability of the applied analytical methods.

### 4.4. Bioaccumulation Factor and Health Risk Assessment

The bioaccumulation factor (BAF) for each element was calculated on a dry matter basis (DM), following the method described by Walker [78]:

$$\mathrm{BAF}=\frac{\mathrm{element}\;\mathrm{concentration}\;\mathrm{in}\;\mathrm{organism}}{\mathrm{element}\;\mathrm{concentration}\;\mathrm{in}\;\mathrm{the}\;\mathrm{diet}}$$

A BAF value exceeding 1 suggests that AD tend to accumulate the respective element.

The hazard quotient (HQ), which serves as an indicator of the non-cancer risk over a lifetime, was calculated by dividing the average daily intake (ADD, mg kg^−1^ day^−1^) by a specific reference dose (RfD, mg kg^−1^ day^−1^), as described by Yang et al. [79] and Girolametti et al. [76,80]:

HQ = ADD/RfD

where ADD is calculated as follows:

ADD = (IR × C)/BW

where IR represents the consumption rate (kg^−1^ day^−1^), C is the average concentration of the metal (mg kg^−1^), and BW is the average body weight (assuming 70 kg). The maximum intake rate for each metal was estimated based on available European dietary surveys from the comprehensive food database [37].

A Hazard Quotient (HQ) ≥ 1 indicates a high risk, while HQ < 1 suggests a low risk.

The Hazard Index (HI) was then calculated by summing the HQ for each PTE:

HI = HQ_Cd_ + HQ_As_ + HQ_Pb_ + HQ_Hg_ + HQ_Ni_ + HQ_Cr_ + HQ_Al_

A HI ≥ 1 indicates a high non-carcinogenic risk, while a value below 1 suggests a low non-carcinogenic risk.

### 4.5. Statistical Analysis

Data are presented as mean ± standard deviation, unless otherwise stated. Statistical analyses were performed using Statgraphics Plus 19 software [81] (Manugistics Inc., Rockville, MD, USA). Comparisons between experimental groups were performed using one-way analysis of variance (ANOVA) followed by the Tukey HSD test at a 95% confidence level. Homogeneity of variance was evaluated using Levene’s test, and when significant variance differences were detected, Welch’s correction was applied to the ANOVA. The correlation between PTE content in the diets and in AD was evaluated using the Pearson correlation, considering the overall dataset from both trials.

## 5. Conclusions

This study focused on the determination of the potentially toxic elements Cd, As, Pb, Hg, Ni, Cr, and Al in AD fed diets supplemented with different percentages of the seaweeds PP and AN, in order to assess the chemical risk to human health associated with the use of insect-derived ingredients as a novel food.

Regarding diets, Cd, Pb, As, and Hg concentrations were below the current legal limits for feed (Regulation No 1869/2019/EU) [10], indicating that diets based on PP and AN are safe for animal consumption. The content of most PTEs in AD is influenced by their presence in their diet. Although AD fed the seaweed-enriched diets accumulated As and Hg, the concentrations for the regulated PTEs (Cd, As, Pb, Hg, and Ni) remained well below the legal limits established by EU regulation 1987/2924 and the amending regulation 915/2023 and fall within the safe levels recommended by the Commission Implementing Regulation (EU) 2022/188 [21]. However, considering the level of Ni, Cr, and Al detected in the diets and the levels of Cr and Al in AD, and considering their toxicity, the authors think that it would be interesting to deepen this topic and suggest that the scientific community identify and set specific limits for the content of these PTEs both in feed and food. Furthermore, the analysis of the risk indexes indicated that AD pose no health hazard to consumers. In conclusion, this study provides valuable insights into the presence of PTEs in house crickets and their implications for human health risk assessment, highlighting the importance of such research in advancing knowledge in this field, promoting greater acceptance of entomophagy, and potentially shifting developed countries towards the approach of consumption of sustainable alternative protein sources.

## Figures and Tables

**Table 1:** Potentially toxic element content (mg kg^−1^ DM) in *Palmaria palmata*, *Ascophyllum nodosum* and diet.

Sample	Cd	As	Pb	Hg	Ni	Cr	Al
**Trial 1**							
*P. palmata*	0.28 ± 0.06	0.5 ± 0.1	0.87 ± 0.05	0.0055 ± 0.0001	3.2 ± 0.4	1.75 ± 0.04	445 ± 63
Ctrl-PP	0.123 ± 0.004	0.046 ± 0.007 ^c^	0.281 ± 0.008 ^d^	0.00243 ± 0.00003 ^d^	6.3 ± 0.4 ^a^	4.0 ± 0.2 ^ab^	426 ± 9
PP5	0.121 ± 0.003	0.054 ± 0.008 ^c^	0.50 ± 0.02 ^c^	0.00280 ± 0.00001 ^c^	5.5 ± 0.3 ^b^	4.3 ± 0.1 ^a^	421 ± 27
PP10	0.123 ± 0.002	0.075 ± 0.010 ^b^	0.62 ± 0.02 ^b^	0.00307 ± 0.00009 ^b^	5.5 ± 0.2 ^b^	3.9 ± 0.1 ^b^	412 ± 2
PP20	0.116 ± 0.001	0.111 ± 0.003 ^a^	0.99 ± 0.07 ^a^	0.0038 ± 0.0002 ^a^	4.8 ± 0.1 ^c^	4.2 ± 0.1 ^a^	422 ± 3
**Trial 2**							
*A. nodosum*	0.31 ± 0.0.09	24 ± 2	0.158 ± 0.004	0.028 ± 0.001	0.71 ± 0.09	1.53 ± 0.04	136 ± 24
Ctrl-AN	0.062 ± 0.009 ^c^	0.050 ± 0.004 ^c^	0.320 ± 0.004 ^a^	0.0026 ± 0.0001 ^c^	6.9 ± 0.1 ^a^	3.6 ± 0.1 ^a^	328 ± 6 ^a^
AN20	0.089 ± 0.004 ^b^	0.53 ± 0.02 ^b^	0.335 ± 0.005 ^a^	0.0065 ± 0.0004 ^b^	6.6 ± 0.2 ^a^	3.7 ± 0.3 ^ab^	317 ± 3 ^b^
AN40	0.126 ± 0.013 ^a^	0.90 ± 0.02 ^a^	0.231 ± 0.003 ^b^	0.0113 ± 0.0001 ^a^	5.3 ± 0.4 ^b^	3.2 ± 0.2 ^b^	300 ± 5 ^c^

PP: *Palmaria palmata*. AN: *Ascophyllum nodosum*. Diet with 0% of *P. palmata* (Ctrl-PP) or 0% of *A. nodosum* (Ctrl-AN); diets enriched with 5% (PP5), 10% (PP10), and 20% (PP20) of *P. palmata*; diets enriched with 20% (AN20) and 40% (AN40) of *A. nodosum*. Different letters in the column indicate statistically significant differences between experimental groups of the same trial (*p* < 0.05). Values are presented as mean ± SD (*n* = 9).

**Table 2:** Bioaccumulation factor for *Acheta domesticus* fed test diets in trial 1 and 2, calculated on a dry weight basis.

Sample	Cd	As	Pb	Hg	Ni	Cr	Al
**Trial 1**							
Ctrl-PP	0.99 ± 0.05	2.0 ± 0.6 ^a^	0.24 ± 0.04 ^a^	2.88 ± 0.07 ^a^	0.20 ± 0.02 ^a^	0.14 ± 0.01 ^a^	0.143 ± 0.005 ^a^
PP5	0.97 ± 0.03	1.6 ± 0.4 ^b^	0.12 ± 0.02 ^b^	2.31 ± 0.07 ^b^	0.13 ± 0.01 ^b^	0.073 ± 0.004 ^b^	0.10 ± 0.01 ^b^
PP10	0.86 ± 0.05	1.5 ± 0.2 ^b^	0.16 ± 0.01 ^b^	2.36 ± 0.07 ^b^	0.14 ± 0.01 ^b^	0.07 ± 0.01 ^b^	0.133 ± 0.004 ^a^
PP20	1.09 ± 0.02	1.1 ± 0.1 ^b^	0.12 ± 0.01 ^b^	2.12 ± 0.13 ^b^	0.18 ± 0.01 ^a^	0.058 ± 0.003 ^b^	0.126 ± 0.004 ^a^
**Trial 2**							
Ctrl-AN	1.1 ± 0.2	1.5 ± 0.2 ^a^	0.17 ± 0.03 ^b^	2.60 ± 0.11 ^a^	0.09 ± 0.01 ^b^	0.16 ± 0.01 ^a^	0.05 ± 0.01
AN20	0.94 ± 0.07	0.32 ± 0.03 ^b^	0.20 ± 0.03 ^b^	1.59 ± 0.10 ^b^	0.11 ± 0.01 ^ab^	0.06 ± 0.01 ^b^	0.057 ± 0.003
AN40	1.1 ± 0.2	0.40 ± 0.04 ^b^	0.31 ± 0.06 ^a^	1.25 ± 0.01 ^b^	0.15 ± 0.02 ^a^	0.05 ± 0.01 ^b^	0.081 ± 0.003

AD fed standard diet with 0% of *Palmaria palmata* (Ctrl-PP) and 0% of *Ascophyllum nodosum* (Ctrl-AN); AD fed diets enriched with 5% (PP5), 10% (PP10), and 20% (PP20) of *P. palmata*; AD fed diets enriched with 20% (AN20) and 40% (AN40) of *A. nodosum*. Different letters in the column indicate statistically significant differences between experimental groups of the same trial (*p* < 0.05). Values are presented as mean ± SD (*n* = 9).

**Table 3:** Potentially toxic element content (mg kg^−1^ WW) in *Acheta domesticus* fed diets enriched with *Palmaria palmata* and *Ascophyllum nodosum* and legal limits.

Sample	Cd	As	Pb	Hg	Ni	Cr	Al
**Legal limit**	0.05 (meat) ^§^ ≤0.05 ^×^	0.2 (rice) ^§^	0.1 (meat) ^§^ ≤0.06 ^×^	0.5 (fish) ^§^	30 (seaweed) *	Not reported	Not reported
**Trial 1**							
Ctrl-PP	0.032 ± 0.001 ^b^	0.024 ± 0.007 ^b^	0.018 ± 0.003 ^c^	0.00169 ± 0.00004 ^b^	0.327 ± 0.027 ^a^	0.147 ± 0.005 ^a^	15.9 ± 0.4 ^a^
PP5	0.034 ± 0.001 ^b^	0.024 ± 0.005 ^b^	0.017 ± 0.003 ^c^	0.00174 ± 0.00005 ^b^	0.211 ± 0.005 ^a^	0.086 ± 0.004 ^ab^	10 ± 1 ^c^
PP10	0.050 ± 0.001 ^a^	0.027 ± 0.002 ^a^	0.023 ± 0.002 ^b^	0.00176 ± 0.00002 ^b^	0.187 ± 0.008 ^b^	0.068 ± 0.004 ^b^	13.0 ± 0.4 ^b^
PP20	0.0308 ± 0.0005 ^b^	0.029 ± 0.003 ^a^	0.029 ± 0.002 ^a^	0.00193 ± 0.00006 ^a^	0.204 ± 0.014 ^a^	0.059 ± 0.002 ^b^	12.9 ± 0.4 ^b^
**Trial 2**							
Ctrl-AN	0.017 ± 0.001 ^b^	0.020 ± 0.001 ^c^	0.014 ± 0.003	0.00166 ± 0.00004 ^b^	0.16 ± 0.02	0.146 ± 0.003 ^a^	4.3 ± 0.4
AN20	0.018 ± 0.001 ^b^	0.038 ± 0.003 ^b^	0.015 ± 0.002	0.00229 ± 0.00003 ^a^	0.16 ± 0.01	0.048 ± 0.002 ^b^	4.0 ± 0.2
AN40	0.027 ± 0.003 ^a^	0.071 ± 0.007 ^a^	0.014 ± 0.003	0.0028 ± 0.0002 ^a^	0.17 ± 0.02	0.032 ± 0.003 ^b^	4.9 ± 0.2

AD fed a control diet with the inclusion of 0% of *Palmaria palmata* (Ctrl-PP) and 0% of *Ascophyllum nodosum* (Ctrl-AN); AD fed diets enriched with 5% (PP5), 10% (PP10), and 20% (PP20) of *P. palmata*; AD fed diets enriched with 20% (AN20) and 40% (AN40) of *A. nodosum*. * EU Regulation 1987/2024 [11], ^§^ EU Regulation 915/2023/EU [12]. ^×^ Commission Implementing Regulation (EU) 2022/188 of 10 February 2022 [21]. Different letters in the column indicate statistically significant differences between experimental groups of the same trial (*p* < 0.05). Values are presented as mean ± SD (*n* = 9).

**Table 4:** Hazard Quotient values (mean ± SD) calculated for each potentially toxic element.

PTEs	HQ
Cd	0.008 ± 0.002
As	0.005 ± 0.004
Pb	0.0016 ± 0.0005
Hg	0.0010 ± 0.0006
Ni	0.0031 ± 0.0007
Cr	0.008 ± 0.005
Al	0.014 ± 0.007

**Table 5:** Hazard Index values (mean ± SD) calculated as the sum of potentially toxic elements for *Acheta domesticus* fed diets supplemented with *Palmaria palmata* (trial 1) and *Ascophyllum nodosum* (trial 2).

Sample	HI
**Trial 1**	
Ctrl-PP	0.056 ± 0.008
PP5	0.040 ± 0.005
PP10	0.046 ± 0.007
PP20	0.046 ± 0.007
**Trial 2**	
Ctrl-AN	0.031 ± 0.005
AN20	0.026 ± 0.002
AN40	0.042 ± 0.004

AD fed a control diet with the inclusion of 0% of *Palmaria palmata* (Ctrl-PP) and 0% of *Ascophyllum nodosum* (Ctrl-AN); AD fed diets enriched with 5% (PP5), 10% (PP10), and 20% (PP20) of *P. palmata*; AD fed diets enriched with 20% (AN20) and 40% (AN40) of *A. nodosum*.

**Table 6:** Potentially toxic element average content (min–max) in *Acheta domesticus* fed different diets.

Diet	Cd	As	Pb	Hg	Ni	Cr	Al	References
	mean ± SD (min–max), mg kg^−1^ DM							
PP-enriched diet AN enriched diet	0.108 ± 0.006 (0.069–0.127)	0.15 ± 0.02 (0.08–0.36)	0.08 ± 0.01 (0.05–0.12)	0.0085 ± 0.0003 (0.0065–0.0141)	0.83 ± 0.06 (0.64–1.2)	0.34 ± 0.01 (0.16–0.58)	38 ± 2 (17–61)	This study
ADD, HRD	(0.01–0.04)	(0.01–0.08)	(0.06–0.20)	nd	(0.13–0.32)	(0.68–1.02)	(9.86–12.58)	[49]
-	<0.03	<0.03	<0.03	nd	0.18	0.24	nd	[50]
-	(0.04–0.06)	nd	(0.1–0.14)	nd	(0.5–0.61)	(0.13–0.18)	nd	[51]
F1, F2, F3	(0.020–0.024)	<0.010	(0.078–0.093)	nd	(0.25–0.41)	(0.4–0.99)	nd	[47]
-	(0.015–0.026)	(<0.01–0.96)	(<0.02–0.115)	(0.038–0.041)	(0.14–0.62)	nd	nd	[52]
-	<0.05	<0.05	(0.102–0.155)	<0.05	nd	nd	nd	[53]

nd: not detected. F1, Oat, wheat, barley, turnip rapeseed meal, potato, and fava beans; F2, Wheat, barley, oat, calcium carbonate, textured soy protein granules, vegetable oil, turnip-rapeseed pellets, calcium sodium phosphate, vitamin and trace element supplementation, sodium chloride (salt), and amino acids. Vitamin and trace element supplementation included vitamins A, D3, and E, iron, iodine, copper, manganese, zinc, and selenium; F3, Wheat, turnip-rapeseed meal, potato, fava beans, peas, and barley. ADD, Aromatic-Arboreal diet; HRD, Human Refuse Diet.

**Table 7:** Accuracy test with certified reference material DORM-2 (Dogfish Muscle Certified Reference Material, NCR Canada). Data are expressed in mg kg^−1^.

Element	Analytical Method	Analytical Results	Certified Values	Δ (%)
Cd	GF-AAS	0.044 ± 0.023	0.043 ± 0.008	+2
As	GF-AAS	16.0 ± 1.0	18.0 ± 1.1	−11
Pb	GF-AAS	0.067 ± 0.031	0.065 ± 0.007	+3
Hg	DMA-1	4.54 ± 0.06	4.64 ± 0.26	−2
Ni	GF-AAS	22.3 ± 2.2	19.4 ± 3.1	+15
Cr	GF-AAS	31.4 ± 0.2	34.7 ± 5.5	−9
Al	GF-AAS	11.1 ± 1.9	10.9 ± 1.7	+2

GF-AAS, Graphite Furnace Atomic Absorption Spectrometer; DMA-1, Direct Mercury Analyzer.

## Data Availability

The original contributions presented in this study are included in the article/Supplementary Materials. Further inquiries can be directed to the corresponding author(s).

## Supplementary Materials

The following supporting information can be downloaded at: https://www.mdpi.com/article/10.3390/molecules30193958/s1, Table S1: Concentration (mg kg^−1^ WW, moisture 12%) of Cadmium (Cd), Lead (Pb), Mercury (Hg), Arsenic (As), Nickel (Ni), Chromium (Cr) and Aluminum (Al) in the diets supplemented with *Palmaria palmata* (trial 1) and *Ascophyllum nodosum* (trial 2), and legal limits for feed material and complete feed (relative to a moisture of 12%), according to Directive 2019/1869/EU on undesirable substances in animal feed.
