# Supplementary material for: Novel Food Safety Evaluation: Potentially Toxic Elements in Acheta domesticus (House Cricket) Reared on Seaweed-Enriched Diets
Source: Molecules. 2025 Oct 2;30(19):3958. doi: 10.3390/molecules30193958 (PMC12526370; doi:10.3390/molecules30193958)
Supplement: Supplementary file 1 [file molecules-30-03958-s001.zip › molecules-3884480-supplementary.pdf]

Novel Food Safety Evaluation: Potentially Toxic Elements in *Acheta domesticus* (House Cricket) Reared on Seaweed-Enriched Diets

Behixhe Ajdini[1], Irene Biancarosa[2], Silvia Illuminati[1], Anna Annibaldi[1], Federico Girolametti[1], Matteo Fanelli[3], Lorenzo Massi[1,4], Cristina Truzzi[1*]

[1] Department of Life and Environmental Sciences, Università Politecnica delle Marche, Via Brecce Bianche, 60131 Ancona, Italy; b.ajdini@staff.univpm.it, f.girolametti@staff.univpm.it, s.illuminati@staff.univpm.it, annibaldi@staff.univpm.it, 956820@stud.unive.it, c.truzzi@staff.univpm.it
[2] Department of Marine Biotechnology, Stazione Zoologica "Anton Dohrn", Fano Marine Center, Viale Adriatico 1-N, 61032 Fano, Italy; biancarosairene@gmail.com
[3] Institute for Marine Biological Resources and Biotechnology (IRBIM), National Research Council (CNR), Largo Fiera della Pesca, 60125 Ancona, Italy; matteo.fanelli@irbim.cnr.it
[4] Department of Environmental Sciences, Informatics and Statistics, Università Ca' Foscari Venezia, Via Torino 155, Mestre 30172, Italy; 956820@stud.unive.it
[*] Correspondence: c.truzzi@staff.univpm.it. Tel.: +39 071 2204514

Table S1. Potentially toxic elements content (mg kg^{-1} WW, moisture 12%) in the diets supplemented with *Palmaria palmata* (trial 1) and *Ascophyllum nodosum* (trial 2), and legal limits.

Sample	Cd	As	Pb	Hg	Ni	Cr	Al
**Legal limit***	0.5	2	5	0.1	Not reported	Not reported	Not reported
**Trial 1**							
P. palmata	0.25±0.05	0.4±0.1	0.77±0.04	0.0050±0.0001	2.8±0.4	1.51±0.04	392±55
Ctrl-PP	0.108±0.004	0.040±0.006^{c}	0.247±0.007^{d}	0.00213±0.0003^{d}	5.5±0.4^{a}	3.5±0.2	375±8
PP5	0.106±0.003	0.048±0.007^{c}	0.44±0.02^{c}	0.00246±0.00001^{c}	4.8±0.3^{b}	3.8±0.1	370±24
PP10	0.108±0.002	0.066±0.009^{b}	0.55±0.02^{b}	0.00270±0.00008^{b}	4.8±0.2^{b}	3.4±0.1	363±2
PP20	0.102±0.001	0.098±0.003^{a}	0.87±0.06^{a}	0.0033±0.0002^{a}	4.2±0.1^{c}	3.7±0.1	371±3
**Trial 2**							
A. nodosum	0.28±0.03	21±2	0.140±0.004	0.025±0.001	0.6±0.1	1.35±0.04	120±21
Ctrl-AN	0.055±0.008^{c}	0.044±0.004^{c}	0.282±0.004^{a}	0.00225±0.00008^{c}	6.1±0.1^{a}	3.2±0.1	289±5
AN20	0.078±0.004^{b}	0.47±0.02^{b}	0.295±0.004^{a}	0.0057±0.0004^{b}	5.8±0.2^{a}	3.3±0.3	279±3
AN40	0.111±0.011^{a}	0.79±0.02^{a}	0.203±0.003^{b}	0.00991±0.00019^{a}	4.7±0.4^{b}	2.8±0.2	264±4

AD fed a control diet with the inclusion of 0% of *Palmaria palmata* (Ctrl-PP) and 0% of *Ascophyllum nodosum* (Ctrl-AN); AD fed diets enriched with 5% (PP5), 10% (PP10) and 20% (PP20) of *P. palmata*; AD fed diets enriched with 20% (AN20) and 40% (AN40) of *A. nodosum*.

* Referred to legal limits in complete feed according to Commission Regulation 2019/1869 [10].

Different letters in the column indicate statistically significant differences between groups of the same trial ($p<0.05$).
